# *Candida auris*: Disinfectants and Implications for Infection Control

**DOI:** 10.3389/fmicb.2018.00726

**Published:** 2018-04-12

**Authors:** Tsun S. N. Ku, Carla J. Walraven, Samuel A. Lee

**Affiliations:** ^1^Section of Infectious Disease, New Mexico VA Health Care System, Albuquerque, NM, United States; ^2^Division of Infectious Diseases, Department of Internal Medicine, University of New Mexico Health Science Center, Albuquerque, NM, United States; ^3^College of Pharmacy, University of New Mexico Health Science Center, Albuquerque, NM, United States

**Keywords:** *Candida auris*, disinfectants, antiseptics, biocides, infection control, decolonization, hand hygiene

## Abstract

*Candida auris* is a rapidly emerging pathogen and is able to cause severe infections with high mortality rates. It is frequently misidentified in most clinical laboratories, thus requiring more specialized identification techniques. Furthermore, several clinical isolates have been found to be multidrug resistant and there is evidence of nosocomial transmission in outbreak fashion. Appropriate infection control measures will play a major role in controlling the management and spread of this pathogen. Unfortunately, there are very few data available on the effectiveness of disinfectants against *C. auris*. Chlorine-based products appear to be the most effective for environmental surface disinfection. Other disinfectants, although less effective than chlorine-based products, may have a role as adjunctive disinfectants. A cleaning protocol will also need to be established as the use of disinfectants alone may not be sufficient for maximal decontamination of patient care areas. Furthermore, there are fewer data on the effectiveness of antiseptics against *C. auris* for patient decolonization and hand hygiene for healthcare personnel. Chlorhexidine gluconate has shown some efficacy in *in vitro* studies but there are reports of patients with persistent colonization despite twice daily body washes with this disinfectant. Hand hygiene using soap and water, with or without chlorhexidine gluconate, may require the subsequent use of alcohol-based hand sanitizer for maximal disinfection. Further studies will be needed to validate the currently studied disinfectants for use in real-world settings.

## Introduction

*Candida auris* is an emerging pathogen which has been isolated in several global regions in a short period of time since its initial discovery. It was first isolated in 2009 in Japan from the ear discharge of a hospitalized patient (Satoh et al., 2009). Since then, cases of *C. auris* have been reported in Asia, Africa, the Middle East, Europe, South America, and, recently, North America (Lee et al., 2011; Chowdhary et al., 2013; Magobo et al., 2014; Emara et al., 2015; Calvo et al., 2016; Schelenz et al., 2016; Vallabhaneni et al., 2016; Ben-Ami et al., 2017; Morales-López et al., 2017; Ruiz Gaitán et al., 2017). The reservoir of this pathogen, however, has not been found, although this pathogen has been found almost exclusively in the hospital setting. Furthermore, many of these clinical isolates are found to be resistant to several of the available antifungal agents. From an epidemiological perspective, the isolation of *C. auris* from various global regions does not fit the typical outbreak transmission patterns of most emerging pathogens. Whereas the outbreaks of most emerging pathogens involve spreading outward from one regional epicenter to other global geographic regions, whole genome sequencing analyses of the clinical *C. auris* isolates from different global locations, i.e., Asia, South Africa, and South America, suggest that the emergence of the clonal populations occurred independently (Lockhart et al., 2017) and spread locally within each region (Chowdhary et al., 2014; Calvo et al., 2016).

Of all of the *Candida* species that are known to cause infections in humans, *C. albicans, C. glabrata, C. tropicalis, C. parapsilosis*, and *C. krusei* are the *Candida* species that have been reported to cause over 95% of the cases of invasive candidiasis (Edmond et al., 1999; Wisplinghoff et al., 2004; Falagas et al., 2010; Pfaller et al., 2011; Andes et al., 2016; Strollo et al., 2016). Although *C. albicans* still accounts for the majority of the cases, recent surveillance studies have shown that the incidence of infections with non-albicans *Candida* species is rising (Al-Rawahi and Roscoe, 2013; Montagna et al., 2014; Hii et al., 2015). There are several virulence factors that have been described with *Candida* species, especially *C. albicans*. These include hyphal formation, adherence, phospholipase and proteinase production, and biofilm formation (Ghannoum, 2000; Berman and Sudbery, 2002; Saville et al., 2003; Fanning and Mitchell, 2012; Moyes et al., 2015). Unlike *C. albicans*, observations of clinical isolates of *C. auris* have revealed that they do not germinate (Borman et al., 2017; Larkin et al., 2017). They also exhibit reduced ability to adhere to silicone elastomer, when compared to *C. albicans*. Additionally, they produce phospholipase and proteinase in a strain-dependent manner, with the majority of the strains tested being non-producers. Moreover, in strains that produced phospholipase, the activity of this enzyme tended to be weak (Larkin et al., 2017). An earlier report by Oh et al. (2011) indicated that the *C. auris* clinical isolates from South Korea did not form biofilms. Two subsequent studies, however, demonstrated that other clinical isolates were able to form biofilms, though they were significantly reduced when compared to *C. albicans* (Larkin et al., 2017; Sherry et al., 2017).

Interestingly, one study has observed different clinical isolates of *C. auris* as having one of two growth characteristic phenotypes, i.e., aggregative and non-aggregative. Borman et al. (2017) noted that a proportion of isolates fail to release the daughter cells after budding, thus forming a large aggregate of cells that cannot be physically separated, described as the aggregative phenotype. Pathogenicity experiments using the *Galleria mellonella* infection model performed by these authors demonstrated that the isolates with non-aggregative phenotype are significantly more pathogenic, comparable to that of *C. albicans*, than the isolates with aggregative phenotype (Borman et al., 2017). Although *C. auris* does not share some of the virulence factors found in many *Candida* species, the non-aggregative phenotype, in conjunction with biofilm-forming ability and innate resistance to antifungal agents, may help explain why it is so pathogenic and resilient on environmental surfaces despite disinfection.

The clinical presentation of *C. auris* infection is, in general, similar to that of the clinical presentation of other *Candida* infections. Colonization is the most commonly reported clinical presentation in one report (Schelenz et al., 2016). Candidemia, however, is the most common clinical disease (Schelenz et al., 2016) followed by a wide-range of other healthcare-associated infections, including intravascular catheter infections (Chowdhary et al., 2014; Schelenz et al., 2016; Biswal et al., 2017; Araúz et al., 2018), urinary tract infections (Vallabhaneni et al., 2016; Morales-López et al., 2017; Araúz et al., 2018), pulmonary infections (Chowdhary et al., 2014; Azar et al., 2017), meningitis (Morales-López et al., 2017), osteomyelitis (Morales-López et al., 2017), otomastoiditis (Choi et al., 2017), and surgical wound infections (Schelenz et al., 2016).

The risk factors associated with the development of invasive *C. auris* infections are also similar to those associated with other *Candida* species. Reviews of patient cases with *C. auris* infections revealed that they were often critically ill, had prior antibiotic or antifungal therapy, had the presence of central venous catheters, underwent recent surgery, or were immunocompromised (Calvo et al., 2016; Vallabhaneni et al., 2016; Lockhart et al., 2017). Alarmingly, high mortality rates of invasive infections with *C. auris* have been reported. The crude mortality rate in Venezuela as reported by Calvo et al. (2016) was 28%, all due to candidemia. In India, Chowdhary et al. (2013) noted the mortality rate from candidemia due to C. auris was as high as 50%. During a hospital outbreak in Panama, the in-hospital mortality rate was 78% (Araúz et al., 2018).

From the review of these reports, there are three distinctive characteristics of *C. auris* that have been elucidated. The first is that most clinical laboratories frequently misidentify this pathogen (Chowdhary et al., 2014; Magobo et al., 2014; Calvo et al., 2016; Vallabhaneni et al., 2016; Morales-López et al., 2017). Biochemical-based identification platforms used in many clinical laboratories, such as VITEK 2 and API 20C AUX, have reportedly misidentified *C. auris* isolates as *C. haemulonii*. Other species have also been incorrectly reported, such as *Rhodotorula glutinis, C. famata, C. sake*, and *Saccharomyces cerevisiae*, depending on the system used (Chowdhary et al., 2014; Jeffery-Smith et al., 2018). The accurate identification of this pathogen typically requires the use of matrix-assisted laser desorption ionization-time of flight spectrometry (MALDI-TOF MS), which is typically not readily available in most clinical laboratories, especially in resource-limited regions (Kathuria et al., 2015). There are, however, other less expensive molecular testing platforms which are currently being investigated for clinical use (Kordalewska et al., 2017; Leach et al., 2018). Until there are updates in the libraries of the biochemical-based identification systems, it will require the acumen of astute clinicians to request further testing of uncommon *Candida* species or unusual yeasts that are found to be multidrug resistant in order for it to be accurately identified.

The second feature is that invasive *C. auris* infections pose a therapeutic challenge owing to the unpredictable antifungal resistance profile that often accompanies this organism. Currently, only three classes of systemic antifungals are available for the treatment of invasive *Candida* infections: polyenes, triazoles, and echinocandins. The polyenes exert their mechanism of action by binding to ergosterol in the fungal cell membrane, causing depolarization and ultimately cell lysis. The triazoles are indirect inhibitors of ergosterol biosynthesis. They inhibit the cytochrome P450 enzyme, lanosterol-14-α-demethylase, which converts the precursor lanosterol to ergosterol. This leads to membrane instability and ultimately cell death. The echinocandins target a unique biosynthesis pathway from polyenes and triazoles. Echinocandins bind to the Fks subunit which blocks β-(1,3)-D-glucan synthesis, which prevents the cross-linking of glucans with other membrane proteins, resulting in the loss of structural integrity of the cell.

Because there are no established species-specific clinical breakpoints or epidemiological cutoff values for *C. auris*, the values established by the Clinical and Laboratory Standards Institute (CLSI) and the European Committee on Antimicrobial Susceptibility Testing (EUCAST) for *C. albicans* have been used for comparison (Clinical and Laboratory Standards Institute, 2017; European Committee on Antimicrobial Susceptibility Testing, 2018). There are, however, some differences in the established clinical breakpoints between both organizations. Although both have defined fluconazole susceptibility as a minimum inhibitory concentration (MIC) ≤ 2 mg/L, resistance is defined as MIC > 8 mg/L, by CLSI and MIC > 4 mg/L by EUCAST. For voriconazole, CLSI has defined susceptibility and resistance of voriconazole as MIC ≤ 0.125 mg/L and MIC > 1 mg/L, respectively, whereas EUCAST defined susceptibility and resistance as MIC ≤ 0.064 mg/L and MIC > 0.25 mg/L, respectively. For all echinocandins, susceptibility and resistance are defined by CLSI as MIC ≤ 0.25 mg/L and MIC ≥ 1 mg/L, respectively. EUCAST, on the other hand, has defined different clinical breakpoints for each of the echinocandins. For anidulafungin and micafungin, susceptible and resistant MIC are defined as 0.032 and 0.016 mg/L, respectively. An isolate is considered susceptible to caspofungin if it is susceptible to anidulafungin. Although there are no established CLSI breakpoints for the polyenes (NCCLS, 2002), EUCAST has defined susceptibility to amphotericin B as ≤1 mg/L.

Epidemiologic studies of clinical isolates, predominately from the blood, have consistently demonstrated elevated MIC to fluconazole (Kathuria et al., 2015; Calvo et al., 2016; Vallabhaneni et al., 2016; Arendrup et al., 2017; Lockhart et al., 2017). Lockhart et al. (2017) found that 41% of *C. auris* isolates in their surveillance study were resistant to at least 2 antifungal classes and 4% were resistant to all three antifungal drug classes. A few studies have suggested that intrinsic fluconazole resistance might be prevalent in *C. auris*, based on recurrent and breakthrough infections when patients were treated with fluconazole (Kathuria et al., 2015). The susceptibility patterns to other triazoles are variable. In one study of 123 clinical *C. auris* isolates, posaconazole (0.015–8 mg/L) and isavuconazole (0.015–4 mg/L) exhibited the lowest MIC, followed by itraconazole (0.032–16 mg/L) (Arendrup et al., 2017). Voriconazole exhibits variable susceptibility to *C. auris* strains (0.032–16 mg/L) (Kathuria et al., 2015; Arendrup et al., 2017). In another study, however, *C. auris* bloodstream isolates from 18 critically ill patients showed a mean voriconazole MIC of 4 mg/L which is higher than the CLSI breakpoint for other *Candida* species (≥2 mg/L) (Lockhart et al., 2017).

Resistance to amphotericin B, a polyene, and echinocandins has also been identified. *C. auris* susceptibilities to amphotericin B are variable in the literature, ranging from 0.25 to 8 mg/L in two retrospective studies (Kathuria et al., 2015; Arendrup et al., 2017). In other studies, the reported MIC range for amphotericin B was lower, 0.28–4 mg/L, but up to one–third of isolates had MIC ≥ 2 mg/L (Calvo et al., 2016; Lockhart et al., 2017). Echinocandin resistance appears to occur at much lower rates than that seen with other antifungal agents. In two studies, clinical isolates against micafungin exhibited the lowest mean MIC of 0.12 mg/L followed by anidulafungin with mean MIC of 0.25 mg/L (Arendrup et al., 2017; Lockhart et al., 2017). It was also reported, however, that seven clinical isolates had elevated echinocandin MIC ≥ 4 mg/L (Arendrup et al., 2017).

The third feature of *C. auris*, which is uncommon among other *Candida* species, is related to how it is spread. The transmission of *C. auris* in healthcare settings has been well documented (Calvo et al., 2016; Schelenz et al., 2016; Vallabhaneni et al., 2016; Biswal et al., 2017; Araúz et al., 2018). Moreover, a donor-derived transmission in a lung transplant recipient was recently described (Azar et al., 2017). The frequent misidentification of this pathogen by many clinical laboratories poses a significant infection control dilemma. The primary concern is the delay in identification of *C. auris* which may result in the delay in the implementation of the appropriate infection control measures to prevent further spread of this pathogen within the healthcare facility.

## Infection Control of *C. auris*

### Role of Environmental Contamination and Patient Colonization in the Transmission of *C. auris*

It has been well established that contaminated environmental surfaces play an important role in the transmission of infectious diseases in the healthcare setting (Weber et al., 2013). Moreover, despite routine cleaning, persistent contamination can occur because of some microorganisms’ ability to form biofilms. In addition to reduced susceptibility to antimicrobial agents and biocides, biofilms also protect microorganisms from hostile environments, even from dehydration for extended periods of time (Lindsay and von Holy, 2006; Smith and Hunter, 2008; Smith et al., 2009). As a result, the proper management of healthcare environmental surfaces is an integral part in the infection control of transmissible diseases (Donskey, 2013).

There are several reports of nosocomial transmission, within and between facilities, of *C. auris* (Calvo et al., 2016; Vallabhaneni et al., 2016; Biswal et al., 2017; Araúz et al., 2018). Furthermore, surveillance studies have shown that *C. auris* can be isolated from environmental surfaces in healthcare facilities (Calvo et al., 2016; Schelenz et al., 2016; Vallabhaneni et al., 2016). Experiments have shown that *C. auris* can survive on and be cultured from surfaces, both moist and dry, for at least 14 days (Piedrahita et al., 2017; Welsh et al., 2017). Additionally, *C. auris* has been cultured from contaminated bedding for up to 7 days (Biswal et al., 2017).

The persistence of this pathogen on environmental surfaces presents opportunities to colonize or infect hospitalized patients and healthcare workers. There is some evidence that skin colonization of *C. auris* can persist for weeks to months (Vallabhaneni et al., 2016). In turn, the shedding of this pathogen from colonized patients and healthcare workers also presents further opportunities for it to contaminate other environmental surfaces. A root cause analysis performed during the first outbreak in a United Kingdom hospital showed that the minimal contact time required for the acquisition of *C. auris* is 4 h (Schelenz et al., 2016). As a result, infection control practitioners and healthcare epidemiologists are targeting both colonized patients and contaminated surfaces as part of their infection control measures.

### Recommendations From World Healthcare Organizations

Presently, there is no established environmental cleaning method in controlling the spread of *C. auris* within healthcare facilities. Many of the major health organizations have issued guidelines and recommendations in regards to the management of *C. auris*, which are listed in **Table 1**. The Centers for Disease Control and Prevention (CDC) recommends the use of the United States Environmental Protection Agency (EPA)-registered hospital-grade disinfectant effective against *Clostridium difficile* spores for the disinfection of surfaces contaminated with *C. auris*. Public Health England (PHE) recommends products containing hypochlorite at 1000 ppm for all cleaning, even if other products are used, e.g., gaseous hydrogen peroxide or UV-C light. The European Centre for Disease Prevention and Control (ECDC) recommends terminal cleaning “using disinfectants and methods with certified antifungal activity”. Both the Public Health Agency of Canada (PHAC) and the South African Centre for Opportunistic, Tropical and Hospital Infections (COTHI), released interim recommendations on the management of *C. auris* infection which suggest “regular” and terminal cleanings with a chlorine-releasing agent at 1000 ppm. Additionally, COTHI suggests the addition of hydrogen peroxide vapor, when feasible. Finally, the Pan American Health Organization/World Health Organization (PAHO/WHO) recommends cleaning with “soap and water followed by disinfection with 0.1% bleach.”

**Table 1:** Recommendations from major world health organizations for infection control of *Candida auris*.

Health Organization	Environmental disinfection	Decolonization procedure	Hand hygiene procedure	Link
Centers for Disease Control and Prevention	Daily and terminal cleaning with use of an EPA-registered hospital-grade disinfectant effective against *C. difficile* spores.	No recommendations.	Use alcohol-based hand sanitizer or hand washing with soap and water, before and after donning gloves.	https://www.cdc.gov/fungal/diseases/candidiasis/c-auris-infection-control.html https://www.epa.gov/pesticide-registration/list-k-epas-registered-antimicrobial-products-effective-against-clostridium
Public Health England	Terminal cleaning with use of a hypochlorite at 1000 ppm. Equipment should be cleaned according to manufacturer’s instructions.	No recommendations.	Hand washing with soap and water followed by alcohol-based hand sanitizer on dried hands, before and after donning gloves.	https://www.gov.uk/government/publications/candida-auris-laboratory-investigation-management-and-infection-prevention-and-control
European Centre for Disease Prevention and Control	Terminal cleaning with disinfectants with certified antifungal activity.	No recommendations.	No recommendations.	https://ecdc.europa.eu/en/publications-data/candida-auris-healthcare-settings
Centre for Opportunistic, Tropical and Hospital Infections (South Africa)	Regular and terminal cleaning with chlorine-releasing agent at 1000 ppm. Consider hydrogen peroxide vapor in terminal cleaning, if feasible.	Not recommended due to limited evidence.	Hand washing with soap and water, especially with soiling, followed use of alcohol-based hand sanitizer.	http://www.nicd.ac.za/assets/files/2016-12-22%20InterimNICDRecommdtnsCAuris.pdf
Pan American Health Organization/World Health Organization	Daily and terminal cleaning with soap and water followed by 0.1% bleach. Clean, disinfect, or sterilize equipment and appliances as per the type of material, after use by the patient. Machine wash linens and clothes.	No recommendations.	No recommendations.	http://www.paho.org/hq/index.php?option=com_docman&task=doc_view&Itemid=270&gid=36354&lang=en

## Disinfection of *C. auris*

### Disinfectants for the Decontamination of Environmental Surfaces

The available data are limited regarding the most effective products and methods for the disinfection of environmental surfaces contaminated by *C. auris*. Because many of these studies use different experimental techniques, the results cannot be readily compared to each other. Furthermore, the results cannot be directly translated to efficacy in real-world scenarios. **Table 2** lists the biocides and disinfectants that have been studied to disinfect surfaces that have been contaminated by *C. auris*. Many of these agents have been first tested against other *Candida* species, specifically sodium hypochlorite, hydrogen peroxide, ethanol, and sodium dodecyl sulfate. In one study, researchers examined the efficacy of a variety of disinfectants and antiseptics against several clinical isolates of *Candida* species from hospitalized patients (Silverman et al., 1999). The authors concluded that a phenol-based and quaternary ammonium detergent were most effective at inhibiting growth of all species at all concentrations and contact time, but all other agents tested were found to be variable in their efficacy. This study, however, was limited as it was performed exclusively on planktonic cells and did not study biofilms. Gupta et al. (2002) also performed experiments whose results supported those findings in both cell suspension tests and directly spraying on Sabouraud’s agar medium plates inoculated with *Candida* species. These authors, however, also found that 1% chlorine, as sodium hypochlorite, was also effective against all *Candida* species tested, in both planktonic and biofilm forms (Gupta et al., 2002).

**Table 2:** Surface disinfectants tested against *C. auris*.

Disinfectant	Concentrations tested (contact time in minutes)	Effective	Level of evidence	Comments	Reference
Chlorine	0.39% (1), 0.65% (1), 0.825% (1), 1% (10), 2% (10), 1000 ppm (3, 5, 180, 1800), 10000 ppm (3, 180, 1800)	Yes	Good	Most extensively studied. Can cause ocular irritation, or oropharyngeal, esophageal, and gastric burns. Can corrode metals at concentrations > 500 ppm.	Abdolrasouli et al., 2017; Biswal et al., 2017; Cadnum et al., 2017b; Moore et al., 2017
Hydrogen peroxide	8 g/m^3^ (?), 1.4% (1)	Yes	Moderate		Abdolrasouli et al., 2017; Cadnum et al., 2017b
Hydrogen peroxide+silver nitrate	11% (60)	Yes	Low		Biswal et al., 2017
Phenolics	5% (?)	Yes	Low	Not FDA-approved for use as high-level disinfectant but can be used to preclean before terminal sterilization.	Biswal et al., 2017
Glutaraldehyde	2% (20)	Yes	Low	Expensive and toxic. Should be used for medical equipment cleaning.	Biswal et al., 2017
Alcohols	29.4% (0.5)	Yes	Low	Difficult to achieve prolonged contact time due to rapid evaporation. Flammable. May harden rubber and certain plastic tubing after prolonged and repeated use.	Cadnum et al., 2017b
Acetic acid	>5% (3)	No	Low		Cadnum et al., 2017b
Peracetic acid	2000 ppm (5, 10)	Yes	Low	For medical equipment cleaning. Can corrode certain metals.	Kean et al., 2018
Peracetic acid+hydrogen peroxide+acetic acid	1200 ppm/<1% (3)	Yes	Low		Cadnum et al., 2017b
Quaternary ammonium compounds	2% didecyldimethyl ammonium chloride (60), alkyl dimethyl ammonium chlorides (10), didecyldimethyl ammonium chloride/dimethylbenzyl ammonium chloride (10)	No	Low		Biswal et al., 2017; Cadnum et al., 2017b

Chlorine-based disinfectants, in the forms of sodium hypochlorite (NaOCl) and sodium dichloroisocyanurate, are also commonly used in the healthcare setting for disinfection, especially against multidrug resistant organisms such as methicillin-resistant *Staphylococcus aureus* (MRSA) and carbapenemase-producing *Enterobacteriacea*. They are the most studied for the disinfection against *C. auris* because they have been previously shown to be extremely effective against other *Candida* species. The effectiveness of chlorine-based disinfectant against *C. auris* was first noted during an outbreak in a United Kingdom hospital in 2015. One thousand parts per million (1000 ppm) chlorine-based product (Chlor-Clean, Guest Medical) was used in the daily routine disinfection of the patient care areas and equipment, and 10000 ppm chlorine-based product (Haz-Tab, Guest Medical) was used for terminal cleaning followed by further disinfection with hydrogen peroxide vapor (Schelenz et al., 2016). Decontamination of the patient care areas was reported to be confirmed by environmental surveillance cultures.

*In vitro* studies have also confirmed the efficacy of chlorine-based disinfectants. Moore et al. evaluated the effectiveness of a commonly used chlorine-based disinfectant (Haz-Tab) by performing a quantitative suspension test of different clinical isolates of *C. auris* treated with 1000 ppm chlorine. With an exposure time of 5 min, all isolates had at least a 4.5 log_10_ reduction in growth (Moore et al., 2017). The authors chose this exposure, or contact time, based on the European Standard (EN 13624:2013) to determine the fungicidal efficacy of chemical disinfectants for quality assurance by manufacturers. In another study, Abdolrasouli et al. (2017) evaluated chlorine-based products at 1000 ppm (Chlor-Clean) and 10000 ppm (Haz-Tab) using a microdilution method against clinical isolates of *C. auris* and other *Candida* species. All isolates were effectively killed at all concentrations with a minimum of 3-min contact time with the exception of *C. parapsilosis* (Abdolrasouli et al., 2017).

Several studies also evaluated the effectiveness of chlorine-based disinfectants on surfaces. Biswal et al. (2017) have tested the effectiveness of 1 and 2% NaOCl solutions applied on four different surfaces (stainless steel, ceramic, plastic, and glass) by inoculating these surface materials with aliquots of *C. auris* cell suspension that were allowed to dry then followed by the application of the disinfectants for a 10-min contact time. They reported complete eradication of *C. auris* on all surfaces (Biswal et al., 2017). In another study, Cadnum et al. (2017b) tested a variety of commercially available healthcare disinfectants, including products containing NaOCl, with concentrations ranging from 0.39 to 0.825%, against *C. auris*. The products were tested in accordance to the American Society for Testing and Materials (ASTM) standard quantitative carrier disk test method (ASTM E2197-11). For the chlorine-based products, all concentrations tested were considered effective against *C. auris* as they all resulted in a ≥5 log_10_ reduction in CFU (Cadnum et al., 2017b). Recently, Kean et al. (2018) tested NaOCl at 1000 and 10000 ppm on cellulose matrix, stainless steel and polyester inoculated with clinical isolates of *C. auris*. NaOCl at all concentrations demonstrated significant killing on all substrates at contact times of 5 and 10 min. However, of all materials tested, complete eradication was achieved only on cellulose substrates (Kean et al., 2018).

Hydrogen peroxide vapor has been used as an adjunct during the terminal cleaning of patient rooms and equipment during the United Kingdom hospital outbreak. Although its activity against *C. auris* had not been previously established at that time, the authors concluded that, in conjunction with using 10000 ppm chlorine-based disinfectant, it effectively decontaminated the environment (Schelenz et al., 2016).

*In vitro* studies have confirmed the killing efficacy of hydrogen peroxide against *C. auris*. In one study, desiccated aliquots of *C. auris* cell suspension in 96-well flat-bottom microtiter plates were exposed to dry gas-vaporized hydrogen peroxide (8 g peroxide/m^3^). These authors reported 96.6–100% killing of the *C. auris* isolates (Abdolrasouli et al., 2017). In another study, 0.5 and 1.4% hydrogen peroxide solutions (Oxivir^®^ Tb and Clorox Healthcare^®^ Hydrogen Peroxide Cleaner Disinfectant) were also found to be effective. The killing efficacy was noted to be comparable to that of chlorine-based disinfectants. Interestingly, the authors noted that the 1.4% hydrogen peroxide disinfectant was effective with 1 min of contact time, which is less than the 3 min recommended by the manufacturer. The 0.5% hydrogen peroxide disinfectant, however, still required the recommended contact time of 10 min for effective killing (Cadnum et al., 2017b). A formulation of 11% stabilized hydrogen peroxide with 0.01% silver nitrate (Ecoshield^®^, Johnson and Johnson) was also found to be effective but it required the 60-min contact time as recommended by the manufacturer for complete eradication (Biswal et al., 2017).

Quaternary ammonium compounds are widely used as disinfectants in many healthcare facilities. They are reported to have fungicidal, bactericidal, and virucidal (against lipophilic viruses) activity and considered to be good cleaning agents. Despite the findings by the two previous studies, a recent study demonstrated them to be ineffective against *Candida* species, including *C. auris* (Cadnum et al., 2017b).

Other disinfectants that have been evaluated against *C. auris* include alcohol, peracetic acid, acetic acid, phenol (phenolic acid), and glutaraldehyde. Two percent glutaraldehyde and 5% phenol were found to be effective on multiple surfaces when the recommended contact times of 20 and 60 min, respectively, were used (Biswal et al., 2017). Ethyl alcohol 29.4% (Purell^®^ Healthcare Surface Disinfectant) was shown to have some killing activity but not to the degree as chlorine-based disinfectants or hydrogen peroxide (Cadnum et al., 2017b). Peracetic acid at 2000 ppm was also found to have significant killing activity against *C. auris*. Like NaOCl, complete eradication was achieved on cellulose matrix but not with steel or plastic (Kean et al., 2018). Furthermore, peracetic acid at 1200 ppm with hydrogen peroxide and acetic acid (OxyCide^TM^ Daily Disinfectant Cleaner) showed killing activity similar to chlorine-based disinfectants (Cadnum et al., 2017b).

In addition to chemical disinfectants, ultraviolet light was also examined to determine its efficacy against *C. auris*. Stainless steel carrier disks inoculated with *Candida* species, including *C. auris, C. difficile* and MRSA were exposed to ultraviolet-C (UV-C) light at 254 nm at varying time durations. UV-C light was most effective against only MRSA at all time durations but significant killing of *C. auris* required at least 20 min of UV-C light exposure (Cadnum et al., 2017a). Furthermore, it was also found that the larger the area the inoculum was spread over, the more effective the UV-C light killing. The authors thus concluded that *C. auris* and other *Candida* species were less effectively killed by UVC light when compared to MRSA but longer exposure may have some benefit as an adjunct to standard cleaning.

### Disinfectants for Decolonization and Hand Hygiene

Since *C. auris* colonization has been recognized as a potential mode of transmission in the healthcare setting, efforts are also focused on decolonization of patients. The goal of decolonization is to reduce, if not eliminate, the microbial load on the patient’s body in order to reduce the risk of infection and transmission. Present decolonization efforts are primarily targeted toward patients who are at risk for infection, specifically surgical and ICU patients, as they are at higher risk of infection (Septimus and Schweizer, 2016). Although there are studies which show that patients with *Staphylococcus aureus* tended to be recolonized after decolonization efforts (Holton et al., 1991; Immerman et al., 2012), it is not clear if this holds true for *C. auris* especially since we have yet to identify the source of *C. auris* that led to the reported outbreaks. **Table 3** lists the antiseptics that have been studied thus far.

**Table 3:** Antiseptics tested against *C. auris*.

Disinfectant	Concentrations tested (contact time in minutes used)	Effective	Level of Evidence	Comments	Reference
Chlorhexidine gluconate	<0.02% (1440), 0.5% (0.5), 2% (2), 4% (3, 180, 1800)	Yes	Good	Most studied antiseptic. Limited clinical evaluation.	Schelenz et al., 2016; Abdolrasouli et al., 2017; Moore et al., 2017; Sherry et al., 2017
Chlorhexidine gluconate in isopropyl alcohol	2%/70% (2)	Yes	Low	*In vitro* testing only.	Moore et al., 2017
Povidone-iodine	10% (2, 3, 180, 1800)	Yes	Moderate	*In vitro* testing only.	Abdolrasouli et al., 2017; Moore et al., 2017;
Alcohol	70%	Yes	Low	Limited clinical evaluation.	Biswal et al., 2017

Chlorhexidine gluconate (CHG), a commonly used antiseptic, is the most studied antiseptic against *C. auris*. It is frequently used in decolonization of patients as well as added to hand soaps in healthcare settings. CHG has been reported to be effective against *Candida* species in several studies (Fathilah et al., 2012; Salim et al., 2013; Yildirim et al., 2014; Mozayeni et al., 2015).

There are only a few studies that evaluated the effectiveness of CHG against *C. auris*. Sherry et al. showed that CHG, at a concentration of <0.02% with a contact time of 24 h, was effective in inhibiting the growth of the planktonic cells and biofilms of clinical isolates of *C. auris* (Sherry et al., 2017). These authors used the CLSI reference methods for broth dilution antifungal susceptibility testing for the planktonic cells while the sessile cells were exposed to the disinfectant for 24 h as outlined by the technique described by Pierce et al. (2008). Abdolrasouli et al. (2017) reported growth inhibition of all clinical isolates of *C. auris* with CHG concentrations between 0.125% to 1.5% with 3-min contact time and increased efficacy at 3 and 30 h. They, however, observed that all clinical isolates of *C. auris* had consistently higher MIC when compared to all other *Candida* species tested, except *C. parapsilosis* (Abdolrasouli et al., 2017). Interestingly, Moore et al. (2017) reported that 2% CHG in 70% isopropyl alcohol was more effective in killing *C. auris* than 2% CHG alone, both with a 2-min contact time. The authors used 4% CHG that is diluted 1:1 with water to produce a 2% CHG solution in order to simulate the addition of tap water during hand washing (Moore et al., 2017).

The two *C. auris* outbreak reports pointed out that despite efforts with twice daily 2% CHG body washes, some patients remained colonized. In one report, however, the authors indicated that some patients had persistent colonization because they were not able to eliminate colonization of the gut, as the patients had diarrhea (Biswal et al., 2017). In the other report, the authors suspected that persistent colonization was due to reinfection from contaminated bedding and clothing. They also considered the possibility of evolving resistance to CHG which they were investigating at the time the report was published (Schelenz et al., 2016).

Iodophors, such as povidone-iodine, are used in skin disinfection preoperatively and in preparation for blood draws that require sterile technique, i.e., for blood cultures. There are only two studies, both *in vitro*, that evaluated povidone-iodine against *C. auris*. Abdolrasouli et al. (2017) reported that the growth of all clinical *C. auris* isolates was inhibited at concentrations between 0.07 and 1.25%, which is below many of the commercially available concentration of 10%, with a minimum contact time of 3 min (Abdolrasouli et al., 2017). Moore et al. concluded that 10% povidone-iodine was also effective against all clinical isolates of *C. auris* with a 2-min contact time (Moore et al., 2017).

There are no published studies that directly evaluate alcohol as an antiseptic against *C. auris*. As previously mentioned, isopropyl alcohol seemed to enhance the disinfection efficacy of CHG (Moore et al., 2017). It was suggested, however, that it was the lack of the adjuvant use of alcohol-based hand rub after routine hand washing by a healthcare personnel that led to colonization by *C. auris* during the United Kingdom hospital outbreak (Schelenz et al., 2016).

## Discussion

Given its virulence, resistance to multiple antifungal agents, high mortality rate, and propensity to colonize patients as well as contaminate environmental surfaces, *C. auris* has become a formidable emerging pathogen. Diligent efforts in infection control to prevent its spread are equally as important as selecting the appropriate antifungal regimen in treating persons with *C. auris* infections. Disinfection and decontamination of the healthcare environment, appropriate hand hygiene of healthcare workers, and decolonization of patients with *C. auris* are integral parts for the effective infection control of *C. auris*. As studies have shown, the choice of biocides and disinfectants is important as commonly used products that are found to be effective against other pathogens may not be as effective against *C. auris*. This is certainly the case with quaternary ammonium compounds which have good activity against MRSA but poor activity against *Candida* species, including *C. auris* (Cadnum et al., 2017b).

Frequently, healthcare facilities choose disinfectants based on their cost, ease of use, the lack of preparations needed, noxiousness to the environmental management and healthcare personnel, and patients, and short contact times. There are, however, other factors that may need to be considered as suggested in some studies.

The type of surface and material may play a significant role in adequate disinfection. Biswal et al. (2017) reported that all of the disinfectants, including 1% NaOCl, were effective on all of the surfaces tested when using the manufacturer recommended 10-min contact. Kean et al. (2018) on the other hand, indicated that there are discrepancies in the effectiveness of disinfection on different hard surface materials, specifically steel and plastic. As complete eradication occurred on a porous surface, as represented by cellulose matrix, these authors speculated that biofilm formation on steel and plastic accounts for the decreased activity of the disinfectants tested. Both studies examined NaOCl at 10000 ppm with similar experimental methods, but the first study used a contact time of 10 min whereas the second study used only 5 min. Thus, the differences in the contact times may account for the differences in results. In addition to the killing efficacy of disinfectants on certain surface materials, material reactivity is also of concern. NaOCl, above 500 ppm, and peracetic acid can be corrosive to certain metals. Since most healthcare environments and equipment contain a combination of materials, this may also pose a disinfection challenge for which limited data exist to guide infection control practices.

The necessary contact time in order for a disinfectant to be effective is also an important factor to consider. Many of these studies exposed *C. auris* to disinfectants over a wide span of contact times, ranging from 30 s to 30 h. It is difficult, as a result, to directly compare the results of the different studies due to varying contact times and different experimental methods used. As many of these studies have shown, the appropriate contact time must be applied depending on the disinfectant. In general, for most disinfectants, the longer the contact time, the more effective the killing efficacy. For the three chlorine-based products tested whose concentrations range from 0.39 to 0.825%, or about 3900–8250 ppm, respectively, the shortest effective killing contact time was 1 min (Cadnum et al., 2017b). For NaOCl concentrations of 1000 and 10000 ppm, a 3-min contact time was found to be effective (Abdolrasouli et al., 2017). Although it may be tempting to choose the disinfecting product with the higher concentration to ensure maximal killing efficacy, a balance between product concentration and tolerance to the product toxicity by all who are exposed these products as well as noxiousness must be determined.

Another important factor to consider in the infection control of *C. auris* is its ability to form biofilms (Oh et al., 2011; Larkin et al., 2017; Sherry et al., 2017). Microbial biofilms have been suspected to play a large role in the survival and persistence of microbes on environmental surfaces. Biofilm formation involves microbes attaching themselves to surfaces where they grow and produce extracellular polymers that help facilitate attachment and matrix formation. This, in turn, has been shown to alter their phenotype in regards to growth and gene expression (Donlan, 2001). Unlike its planktonic counterpart, one of the cardinal characteristics of biofilms is marked resistance to environmental stressors, such as desiccation, as well as exposure to antifungal agents and biocides (Chandra and Mukherjee, 2015). There are several proposed resistance mechanisms which include alterations in cellular metabolic activities, increased expression of certain drug resistance genes, and interactions of the extracellular polysaccharide matrix with antifungal agents. This phenomenon is also found to be true for biocides and disinfectants. It has been shown that the concentrations of certain biocides, such as ethanol, hydrogen peroxide and sodium dodecyl sulfate, needed to kill *C. albicans* biofilm cells are several-fold higher than that needed to kill their planktonic counterparts (Nett et al., 2008). The exact mechanisms bestowing biocide resistance are unclear but Watamoto et al. (2010) postulated that surface adhesion of cells during the early phase of biofilm formation may induce resistance mechanisms. This was based on their observations that *C. albicans* mutants lacking the filamentation regulating gene *EFG1* were more susceptible to biocides than the wild type strains during the adhesion phase (Watamoto et al., 2010). Moreover, other researchers have reported similar findings (Leung et al., 2012). Additionally, it is known that within biofilms are a sub-population of “persister” cells (Lewis, 2010). These cells are metabolically inert and are consequently more resistant to antifungal agents whose killing mechanisms are dependent upon metabolically active and replicating cells. As a result, the presence of these cells is one of the reasons that chronic infections involving biofilms are extremely difficult to eradicate.

Although the ability of *C. auris* to form biofilms appears to be strain-dependent, there are no reports of *C. auris* isolates collected from environmental surfaces that form biofilms as of yet. Nevertheless, it may be conceivable that the persistence of *C. auris* on environmental surfaces may be due biofilm-forming isolates. Despite studies showing *C. auris* biofilms having significantly reduced susceptibility to most antifungal agents (Larkin et al., 2017; Sherry et al., 2017), there are scarce data on the effectiveness of disinfectants against *C. auris* biofilms. Given the innate resistance of biofilms to biocides, it seems unlikely that the simple direct application of any disinfectant, even if it was found to be effective *in vitro*, will be sufficient to adequately disinfect environmental surfaces contaminated with *C. auris*. Thus, physically dislodging biofilms may be necessary prior to the application of the disinfectant in order to improve its biocidal activity. Many studies have evaluated the effectiveness of the disinfectants on planktonic cells or cells during the adhesion phase of biofilm formation. It is reasonable to assume that a longer contact time may be necessary to allow time for the disinfectants to penetrate the biofilms in order for them to be effective. More studies, however, will be needed to determine the effectiveness of disinfectants against *C. auris* biofilms.

Since there are no registered products specifically for use against *C. auris* and disinfectant testing methods have been variable, the EPA has recently issued an interim guidance on evaluating the efficacy of products against *C. auris*^1^. The testing method is similar to that which was used by Cadnum et al. (2017b) in which stainless steel carrier disks are inoculated, dried, and then treated with the disinfectant. Based on the description of the testing method, the cell suspension inoculum is allowed to dry for about 50 min, thus allowing *C. auris* to develop biofilms and likely to be more representative of how *C. auris* would be found on environmental surfaces in healthcare settings.

At present, the CDC recommends the use of EPA-registered hospital-grade disinfectant effective against *Clostridium difficile* spores. Many of the products on that list are chlorine-based^2^. Both PHE and COTHI also recommend chlorine-based disinfectant for routine daily and terminal cleaning. The ECDC recommends emphasis on terminal cleaning of contaminated rooms using disinfectants and methods with certified antifungal activity, as determined by the European Standard EN 13624:2013. Although all of these guidelines rely on the manufacturer’s recommended contact times, they do not offer specific cleaning method, frequency, or the addition of another disinfectant.

From the U.K. hospital outbreak, thrice daily cleaning with chlorine-based disinfectant at 1000 ppm around patient bed areas, and 10000 ppm for terminal cleaning seems to sufficiently disinfect contaminated areas, as confirmed by environmental surveillance cultures (Abdolrasouli et al., 2017). Although Biswal et al. (2017) reported effective environmental disinfection with a stabilized hydrogen peroxide with silver nitrate product (Ecoshield^®^), they did not indicate the frequency of daily cleaning used. Therefore, more frequent daily cleaning, at least twice daily, may be considered, at least until more data are available. The role of extensive wiping of hard surfaces with a biocide active against *C. auris* should also be further evaluated, as the dislodging of biofilms and removal of debris may improve the actions of disinfectants. Further disinfection, with either hydrogen peroxide vapor or UV-C light, during terminal cleaning may provide some additional benefit, although more data are also needed to validate this practice. Although, as previously noted, *C. auris* has shown relative resistance to UV-C light, this may be related to the testing method as suggested by the authors who performed the experiments (Cadnum et al., 2017a). Thus, further studies on UV-C light are needed to determine the optimal distance from the device and duration of UV-C light exposure for maximum disinfection. Finally, the method for the disinfection of more intricate healthcare equipment, e.g., ECG monitor leads and blood pressure monitors and cuffs, needs to be determined. Disinfection with hydrogen peroxide vapor has been used for this purpose and may also be considered (Abdolrasouli et al., 2017).

There is no established patient decolonization method for *C. auris* at this time. The CDC and ECDC emphasized screening for colonized persons but did not offer a decolonization protocol. PHE recommends body washes and mouth gargles with CHG. It also advocates for the use of topical nystatin and terbinafine for certain key sites such as venous cannula sites. PHE, however, did not provide any specific details, e.g., concentration of CHG, frequency of application, etc. COTHI did not address decolonization in their guidelines. It is unclear if CHG is ineffective as an antiseptic in decolonization of patients with *C. auris*. As reported from the experiences of the outbreaks in the United Kingdom and India, other factors were used to explain the persistent colonization of some patients. Moreover, it may be that the establishment of a more extensive decolonization protocol is needed for better results. Certainly, other antiseptics, or various combinations of existing antiseptics, should be evaluated for efficacy. One consideration of interest is the use of very dilute solutions of household bleach, i.e., NaOCl, as suggested in the guidelines issued by the Infectious Diseases Society of America for the management of MRSA infections for decolonization (Liu et al., 2011). Although povidone iodine has not been studied or used in whole body decolonization methods, studies appear to support its use for antiseptic skin preparation against *C. auris* (Moore et al., 2017). As this is commonly used for preoperative skin disinfection, healthcare facilities may continue to use this product for this reason.

While all of the guidelines emphasize strict adherence to hand hygiene by healthcare personnel, they differ in their recommended methods. The CDC recommends using alcohol-based hand sanitizer, or washing with soap and water if the hands are visibly soiled. The PHE and COTHI recommend washing hands with soap and water followed by the use of alcohol-based hand rubs on dry hands. The ECDC did not recommend any specific hand hygiene method. There are very limited data on the efficacy of disinfectants against *C. auris* on hand hygiene. One study claimed that washing hands with soap and water using the steps recommended by the World Health Organization was as effective as alcohol-based hand sanitizers, with and without CHG, in removing *C. auris* (Biswal et al., 2017). Plain soap, which is primarily a detergent, has no inherent antimicrobial property. When used in conjunction with water, it helps with removing lipids, and dislodging adherent soil and organic substances from skin. Although it may help with dislodging loosely bound microbes, it may not be sufficient to completely eradicate *C. auris* from the hands of healthcare personnel. As recommended by PHE and COTHI, it would be reasonable to follow hand washing with an alcohol-based hand sanitizer. The combination of CHG and alcohol-based hand rub products may also provide some additional benefit as suggested by a study that reported that the addition of isopropyl alcohol enhanced the disinfecting activity of CHG when tested against *Klebsiella pneumoniae* (Bock et al., 2016).

## Conclusion

Many questions exist regarding the best infection control practices for the management of *C. auris*. The development of any effective infection control protocol will certainly involve choosing the best disinfectants for decontamination. Unfortunately, most of the published studies on disinfectants against *C. auris* used different experimental techniques which makes it difficult to compare the results. Furthermore, many of these experiments were not performed in conditions that are representative of the typical patient care environment, including the disinfection of fabrics and other materials commonly found in the healthcare setting. At this time, there is no single disinfectant that has been established to be effective for all surfaces and materials. Thus, the search for newer disinfectants should continue as well as evidence-based decontamination methods. Finally, since patient decolonization and healthcare personnel hand hygiene are equally important for infection control of *C. auris*, further research in other skin disinfectants and decolonization methods will be needed to help establish more effective methods.

## Author Contributions

TK and CW reviewed the literature and drafted the manuscript. SL reviewed and edited the manuscript.

## Conflict of Interest Statement

The authors declare that the research was conducted in the absence of any commercial or financial relationships that could be construed as a potential conflict of interest.

## Footnotes

1 https://www.epa.gov/sites/production/files/2017-03/documents/mb-35-00.pdf

2 https://www.epa.gov/sites/production/files/2018-01/documents/2018.10.01.listk_.pdf

## References

[B1] Abdolrasouli A. Armstrong-James D. Ryan L. Schelenz S. (2017). In vitro efficacy of disinfectants utilised for skin decolonisation and environmental decontamination during a hospital outbreak with *Candida auris*. *Mycoses* 60 758–763. 10.1111/myc.12699 28872735

[B2] Al-Rawahi G. N. Roscoe D. L. (2013). Ten-year review of candidemia in a Canadian tertiary care centre: predominance of non-*albicans Candida* species. *Can. J. Infect. Dis. Med. Microbiol.* 24 e65–e68. 10.1155/2013/929717 24421833 PMC3852460

[B3] Andes D. R. Safdar N. Baddley J. W. Alexander B. Brumble L. Freifeld A. (2016). The epidemiology and outcomes of invasive *Candida* infections among organ transplant recipients in the United States: results of the Transplant-Associated Infection Surveillance Network (TRANSNET). *Transpl. Infect. Dis.* 18 921–931. 10.1111/tid.12613 27643395

[B4] Araúz A. B. Caceres D. H. Santiago E. Armstrong P. Arosemena S. Ramos C. (2018). Isolation of *Candida auris* from 9 patients in Central America: importance of accurate diagnosis and susceptibility testing. *Mycoses* 61 44–47. 10.1111/myc.12709 28945325

[B5] Arendrup M. C. Prakash A. Meletiadis J. Sharma C. Chowdhary A. (2017). Comparison of EUCAST and CLSI reference microdilution MICs of eight antifungal compounds for *Candida auris* and associated tentative epidemiological cutoff values. *Antimicrob. Agents Chemother.* 61:e485-17. 10.1128/AAC.00485-17 28416539 PMC5444165

[B6] Azar M. M. Turbett S. E. Fishman J. A. Pierce V. M. (2017). Donor-derived transmission of *Candida auris* during lung transplantation. *Clin. Infect. Dis.* 65 1040–1042. 10.1093/cid/cix460 28520901

[B7] Ben-Ami R. Berman J. Novikov A. Bash E. Shachor-Meyouhas Y. Zakin S. (2017). Multidrug-resistant *Candida haemulonii* and *C. auris*, Tel Aviv, Israel. *Emerg. Infect. Dis.* 23 195–203. 10.3201/eid2302.161486 28098529 PMC5324804

[B8] Berman J. Sudbery P. E. (2002). *Candida albicans*: a molecular revolution built on lessons from budding yeast. *Nat. Rev. Genet.* 3 918–932. 10.1038/nrg948 12459722

[B9] Biswal M. Rudramurthy S. M. Jain N. Shamanth A. S. Sharma D. Jain K. (2017). Controlling a possible outbreak of *Candida auris* infection: lessons learnt from multiple interventions. *J. Hosp. Infect.* 97 363–370. 10.1016/j.jhin.2017.09.009 28939316

[B10] Bock L. J. Wand M. E. Sutton J. M. (2016). Varying activity of chlorhexidine-based disinfectants against *Klebsiella pneumoniae* clinical isolates and adapted strains. *J. Hosp. Infect.* 93 42–48. 10.1016/j.jhin.2015.12.019 26899354

[B11] Borman A. M. Szekely A. Johnson E. M. (2017). Isolates of the emerging pathogen *Candida auris* present in the UK have several geographic origins. *Med. Mycol.* 55 563–567. 10.1093/mmy/myw147 28204557

[B12] Cadnum J. L. Shaikh A. A. Piedrahita C. T. Jencson A. L. Larkin E. L. Ghannoum M. A. (2017a). Relative resistance of the emerging fungal pathogen *Candida auris* and other *Candida* species to killing by ultraviolet light. *Infect. Control Hosp. Epidemiol.* 39 94–96. 10.1017/ice.2017.239 29157326

[B13] Cadnum J. L. Shaikh A. A. Piedrahita C. T. Sankar T. Jencson A. L. Larkin E. L. (2017b). Effectiveness of disinfectants against *Candida auris* and other *Candida* species. *Infect. Control Hosp. Epidemiol.* 38 1240–1243. 10.1017/ice.2017.162 28793937

[B14] Calvo B. Melo A. S. A. Perozo-Mena A. Hernandez M. Francisco E. C. Hagen F. (2016). First report of *Candida auris* in America: clinical and microbiological aspects of 18 episodes of candidemia. *J. Infect.* 73 369–374. 10.1016/j.jinf.2016.07.008 27452195

[B15] Chandra J. Mukherjee P. K. (2015). *Candida* biofilms: development, architecture, and resistance. *Microbiol. Spectr.* 3:MB-0020-2015. 10.1128/microbiolspec.MB-0020-2015 26350306 PMC4566167

[B16] Choi H. I. An J. Hwang J. J. Moon S. Son J. S. (2017). Otomastoiditis caused by *Candida auris*: case report and literature review. *Mycoses* 60 488–492. 10.1111/myc.12617 28378904

[B17] Chowdhary A. Kumar V. A. Sharma C. Prakash A. Agarwal K. Babu R. (2014). Multidrug-resistant endemic clonal strain of *Candida auris* in India. *Eur. J. Clin. Microbiol. Infect. Dis.* 33 919–926. 10.1007/s10096-013-2027-1 24357342

[B18] Chowdhary A. Sharma C. Duggal S. Agarwal K. Prakash A. Singh P. K. (2013). New clonal strain of *Candida auris*, Delhi, India. *Emerg. Infect. Dis.* 19 1670–1673. 10.3201/eid1910.130393 24048006 PMC3810747

[B19] Clinical and Laboratory Standards Institute (2017). *Performance Standards for Antifungal Susceptibility Testing of Yeasts*, 1st Edn. *CLSI Supplement M60.* Wayne, PA: CLSI.

[B20] Donlan R. M. (2001). Biofilm formation: a clinically relevant microbiological process. *Clin. Infect. Dis.* 33 1387–1392. 10.1086/322972 11565080

[B21] Donskey C. J. (2013). Does improving surface cleaning and disinfection reduce health care-associated infections? *Am. J. Infect. Control* 41(5 Suppl.) S12–S19. 10.1016/j.ajic.2012.12.010 23465603

[B22] Edmond M. B. Wallace S. E. McClish D. K. Pfaller M. A. Jones R. N. Wenzel R. P. (1999). Nosocomial bloodstream infections in United States hospitals: a three-year analysis. *Clin. Infect. Dis.* 29 239–244. 10.1086/520192 10476719

[B23] Emara M. Ahmad S. Khan Z. Joseph L. Al-Obaid I. Purohit P. (2015). *Candida auris* candidemia in Kuwait, 2014. *Emerg. Infect. Dis.* 21 1091–1092. 10.3201/eid2106.150270 25989098 PMC4451886

[B24] European Committee on Antimicrobial Susceptibility Testing (2018). *Antifungal Agents Breakpoint Tables for Interpretation of MICs. European Committee on Antimicrobial Susceptibility Testing 2018.* Available at: http://www.eucast.org/fileadmin/src/media/PDFs/EUCAST_files/AFST/Clinical_breakpoints/Antifungal_breakpoints_v_9.0_180212.pdf

[B25] Falagas M. E. Roussos N. Vardakas K. Z. (2010). Relative frequency of albicans and the various non-albicans Candida spp among candidemia isolates from inpatients in various parts of the world: a systematic review. *Int. J. Infect. Dis.* 14 e954–e966. 10.1016/j.ijid.2010.04.006 20797887

[B26] Fanning S. Mitchell A. P. (2012). Fungal Biofilms. *PLoS Pathog.* 8:e1002585. 10.1371/journal.ppat.1002585 22496639 PMC3320593

[B27] Fathilah A. R. Himratul-Aznita W. H. Fatheen A. R. N. Suriani K. R. (2012). The antifungal properties of chlorhexidine digluconate and cetylpyrinidinium chloride on oral *Candida*. *J. Dent.* 40 609–615. 10.1016/j.jdent.2012.04.003 22521700

[B28] Ghannoum M. A. (2000). Potential role of phospholipases in virulence and fungal pathogenesis. *Clin. Microbiol. Rev.* 13 122–143. 10.1128/CMR.13.1.122-143.2000 10627494 PMC88936

[B29] Gupta A. K. Ahmad I. Summerbell R. C. (2002). Fungicidal activities of commonly used disinfectants and antifungal pharmaceutical spray preparations against clinical strains of Aspergillus and Candida species. *Med. Mycol.* 40 201–208. 10.1080/mmy.40.2.201.208 12058733

[B30] Hii I.-M. Chang H.-L. Lin L.-C. Lee Y.-L. Liu Y.-M. Liu C.-E. (2015). Changing epidemiology of candidemia in a medical center in middle Taiwan. *J. Microbiol. Immunol. Infect.* 48 306–315. 10.1016/j.jmii.2013.08.017 24113067

[B31] Holton D. L. Nicolle L. E. Diley D. Bernstein K. (1991). Efficacy of mupirocin nasal ointment in eradicating *Staphylococcus aureus* nasal carriage in chronic haemodialysis patients. *J. Hosp. Infect.* 17 133–137. 10.1016/0195-6701(91)90177-A 1674259

[B32] Immerman I. Ramos N. L. Katz G. M. Hutzler L. H. Phillips M. S. Bosco J. A. (2012). The persistence of *Staphylococcus aureus* decolonization after mupirocin and topical chlorhexidine: implications for patients requiring multiple or delayed procedures. *J. Arthroplasty* 27 870–876. 10.1016/j.arth.2012.01.010 22397861

[B33] Jeffery-Smith A. Taori S. K. Schelenz S. Jeffery K. Johnson E. M. Borman A. (2018). *Candida auris*: a review of the literature. *Clin. Microbiol. Rev.* 31:e29-17. 10.1128/CMR.00029-17 29142078 PMC5740969

[B34] Kathuria S. Singh P. K. Sharma C. Prakash A. Masih A. Kumar A. (2015). Multidrug-resistant *Candida auris* misidentified as *Candida haemulonii*: characterization by matrix-assisted laser desorption ionization–time of flight mass spectrometry and DNA sequencing and its antifungal susceptibility profile variability by Vitek 2, CLSI broth microdilution, and Etest method. *J. Clin. Microbiol.* 53 1823–1830. 10.1128/JCM.00367-15 25809970 PMC4432077

[B35] Kean R. Sherry L. Townsend E. McKloud E. Short B. Akinbobola A. (2018). Surface disinfection challenges for *Candida auris*: an in vitro study. *J. Hosp. Infect.* 98 433–436. 10.1016/j.jhin.2017.11.015 29203448

[B36] Kordalewska M. Zhao Y. Lockhart S. R. Chowdhary A. Berrio I. Perlin D. S. (2017). Rapid and accurate molecular identification of the emerging multidrug-resistant pathogen *Candida auris*. *J. Clin. Microbiol.* 55 2445–2452. 10.1128/JCM.00630-17 28539346 PMC5527423

[B37] Larkin E. Hager C. Chandra J. Mukherjee P. K. Retuerto M. Salem I. (2017). The emerging pathogen *Candida auris*: growth phenotype, virulence factors, activity of antifungals, and effect of SCY-078, a novel glucan synthesis inhibitor, on growth morphology and biofilm formation. *Antimicrob. Agents Chemother.* 61:e2396-16. 10.1128/AAC.02396-16 28223375 PMC5404565

[B38] Leach L. Zhu Y. Chaturvedi S. (2018). Development and validation of a real-time PCR assay for rapid detection of *Candida auris* from surveillance samples. *J. Clin. Microbiol.* 24:e1223-17. 10.1128/JCM.01223-17 29187562 PMC5786737

[B39] Lee W. G. Shin J. H. Uh Y. Kang M. G. Kim S. H. Park K. H. (2011). First three reported cases of nosocomial fungemia caused by *Candida auris*. *J. Clin. Microbiol.* 49 3139–3142. 10.1128/JCM.00319-11 21715586 PMC3165631

[B40] Leung C. Y. Chan Y. C. Samaranayake L. P. Seneviratne C. J. (2012). Biocide resistance of *Candida* and *Escherichia coli* biofilms is associated with higher antioxidative capacities. *J. Hosp. Infect.* 81 79–86. 10.1016/j.jhin.2011.09.014 22595316

[B41] Lewis K. (2010). Persister cells. *Annu. Rev. Microbiol.* 64 357–372. 10.1146/annurev.micro.112408.134306 20528688

[B42] Lindsay D. von Holy A. (2006). Bacterial biofilms within the clinical setting: what healthcare professionals should know. *J. Hosp. Infect.* 64 313–325. 10.1016/j.jhin.2006.06.028 17046102

[B43] Liu C. Bayer A. Cosgrove S. E. Daum R. S. Fridkin S. K. Gorwitz R. J. (2011). Clinical practice guidelines by the Infectious Diseases Society of America for the treatment of methicillin-resistant *Staphylococcus aureus* infections in adults and children. *Clin. Infect. Dis.* 52 e18–e55. 10.1093/cid/cir034 21208910

[B44] Lockhart S. R. Etienne K. A. Vallabhaneni S. Farooqi J. Chowdhary A. Govender N. P. (2017). Simultaneous emergence of multidrug-resistant *Candida auris* on 3 continents confirmed by whole-genome sequencing and epidemiological analyses. *Clin. Infect. Dis.* 15 134–140. 10.1093/cid/ciw691 27988485 PMC5215215

[B45] Magobo R. E. Corcoran C. Seetharam S. Govender N. P. (2014). *Candida auris*–associated candidemia, South Africa. *Emerg. Infect. Dis.* 20 1250–1252. 10.3201/eid2007.131765 24963796 PMC4073876

[B46] Montagna M. T. Lovero G. Borghi E. Amato G. Andreoni S. Campion L. (2014). Candidemia in intensive care unit: a nationwide prospective observational survey (GISIA-3 study) and review of the European literature from 2000 through 2013. *Eur. Rev. Med. Pharmacol. Sci.* 18 661–674. 24668706

[B47] Moore G. Schelenz S. Borman A. M. Johnson E. M. Brown C. S. (2017). Yeasticidal activity of chemical disinfectants and antiseptics against *Candida auris*. *J. Hosp. Infect.* 97 371–375. 10.1016/j.jhin.2017.08.019 28865738

[B48] Morales-López S. E. Parra-Giraldo C. M. Ceballos-Garzón A. Martínez H. P. Rodríguez G. J. Álvarez-Moreno C. A. (2017). Invasive infections with multidrug-resistant yeast *Candida auris*, Colombia. *Emerg. Infect. Dis.* 23 162–164. 10.3201/eid2301.161497 27983941 PMC5176232

[B49] Moyes D. L. Richardson J. P. Naglik J. R. (2015). *Candida albicans*-epithelial interactions and pathogenicity mechanisms: scratching the surface. *Virulence* 19 338–346. 10.1080/21505594.2015.1012981 25714110 PMC4601190

[B50] Mozayeni M. A. Hadian A. Bakhshaei P. Dianat O. (2015). Comparison of antifungal activity of 2% chlorhexidine, calcium hydroxide, and nanosilver gels against *Candida albicans*. *J. Dent.* 12 109–117. 26056520 PMC4434124

[B51] NCCLS (2002). *Reference Method for Broth Dilution Antifungal Susceptibility of Yeasts; Approved Standard–2nd Edn.* NCCLS document M27-A2. Wayne, PA: NCCLS.

[B52] Nett J. E. Guite K. M. Ringeisen A. Holoyda K. A. Andes D. R. (2008). Reduced biocide susceptibility in *Candida albicans* biofilms. *Antimicrob. Agents Chemother.* 52 3411–3413. 10.1128/AAC.01656-07 18573927 PMC2533490

[B53] Oh B. J. Shin J. H. Kim M.-N. Sung H. Lee K. Joo M. Y. (2011). Biofilm formation and genotyping of *Candida haemulonii, Candida pseudohaemulonii*, and a proposed new species (*Candida auris*) isolates from Korea. *Med. Mycol.* 49 98–102. 10.3109/13693786.2010.493563 20560864

[B54] Pfaller M. A. Moet G. J. Messer S. A. Jones R. N. Castanheira M. (2011). *Candida* bloodstream infections: comparison of species distributions and antifungal resistance patterns in community-onset and nosocomial isolates in the SENTRY Antimicrobial Surveillance Program, 2008-2009. *Antimicrob. Agents Chemother.* 55 561–566. 10.1128/AAC.01079-10 21115790 PMC3028787

[B55] Piedrahita C. T. Cadnum J. L. Jencson A. L. Shaikh A. A. Ghannoum M. A. Donskey C. J. (2017). Environmental surfaces in healthcare facilities are a potential source for transmission of *Candida auris* and other *Candida* species. *Infect. Control Hosp. Epidemiol.* 38 1107–1109. 10.1017/ice.2017.127 28693657

[B56] Pierce C. G. Uppuluri P. Tristan A. R. Jr. Flw Mowat E. Ramage G. (2008). A simple and reproducible 96-well plate-based method for the formation of fungal biofilms and its application to antifungal susceptibility testing. *Nat. Protoc.* 3 1494–1500. 10.1038/nport.2008.141 18772877 PMC2741160

[B57] Ruiz Gaitán A. C. Moret A. López Hontangas J. L. Molina J. M. Aleixandre López A. I. Cabezas A. H. (2017). Nosocomial fungemia by *Candida auris*: first four reported cases in continental Europe. *Rev Iberoam Micol.* 34 23–27. 10.1016/j.riam.2016.11.002 28131716

[B58] Salim N. Moore C. Silikas N. Satterthwaite J. Rautemaa R. (2013). Chlorhexidine is a highly effective topical broad-spectrum agent against *Candida* spp. *Int. J. Antimicrob. Agents* 41 65–69. 10.1016/j.ijantimicag.2012.08.014 23084595

[B59] Satoh K. Makimura K. Hasumi Y. Nishiyama Y. Uchida K. Yamaguchi H. (2009). *Candida auris* sp. nov., a novel ascomycetous yeast isolated from the external ear canal of an inpatient in a Japanese hospital. *Microbiol. Immunol.* 53 41–44. 10.1111/j.1348-0421.2008.00083.x 19161556

[B60] Saville S. P. Lazzell A. L. Monteagudo C. Lopez-Ribot J. L. (2003). Engineered control of cell morphology in vivo reveals distinct roles for yeast and filamentous forms of *Candida albicans* during Infection. *Eukaryot. Cell* 2 1053–1060. 10.1128/EC.2.5.1053-1060.2003 14555488 PMC219382

[B61] Schelenz S. Hagen F. Rhodes J. L. Abdolrasouli A. Chowdhary A. Hall A. (2016). First hospital outbreak of the globally emerging *Candida auris* in a European hospital. *Antimicrob. Resist. Infect. Control* 5:35. 10.1186/s13756-016-0132-5 27777756 PMC5069812

[B62] Septimus E. J. Schweizer M. L. (2016). Decolonization in prevention of health care-associated infections. *Clin. Microbiol. Rev.* 29 201–222. 10.1128/CMR.00049-15 26817630 PMC4786886

[B63] Sherry L. Ramage G. Kean R. Borman A. Johnson E. M. Richardson M. D. (2017). Biofilm-forming capability of highly virulent, multidrug-resistant *Candida auris*. *Emerg. Infect. Dis.* 23 328–331. 10.3201/eid2302.161320 28098553 PMC5324806

[B64] Silverman J. Vazquez J. A. Sobel J. D. Zervos M. J. (1999). Comparative in vitro activity of antiseptics and disinfectants versus clinical isolates of *Candida* species. *Infect. Control Hosp. Epidemiol.* 20 676–684. 10.1086/501564 10530645

[B65] Smith K. Hunter I. S. (2008). Efficacy of common hospital biocides with biofilms of multi-drug resistant clinical isolates. *J. Med. Microbiol.* 57 966–973. 10.1099/jmm.0.47668-0 18628497

[B66] Smith K. Perez A. Ramage G. Gemmell C. G. Lang S. (2009). Comparison of biofilm-associated cell survival following in vitro exposure of meticillin-resistant *Staphylococcus aureus* biofilms to the antibiotics clindamycin, daptomycin, linezolid, tigecycline and vancomycin. *Int. J. Antimicrob. Agents* 33 374–378. 10.1016/j.ijantimicag.2008.08.029 19101124

[B67] Strollo S. Lionakis M. S. Adjemian J. Steiner C. A. Prevots D. R. (2016). Epidemiology of hospitalizations associated with invasive candidiasis, United States, 2002–2012. *Emerg. Infect. Dis.* 23 7–13. 10.3201/eid2301.161198 27983497 PMC5176241

[B68] Vallabhaneni S. Kallen A. Tsay S. Chow N. Welsh R. Kerins J. (2016). Investigation of the first seven reported cases of *Candida auris*, a globally emerging invasive, multidrug-resistant fungus - United States, May 2013-August 2016. *MMWR Morb. Mortal. Wkly. Rep.* 11 1234–1237. 10.15585/mmwr.mm6544e1 27832049

[B69] Watamoto T. Samaranayake L. P. Egusa H. Yatani H. Samaranayke Y. H. Seneviratne C. J. (2010). Susceptibility of *Candida albicans* filamentation-defective mutants to clinical biocides. *J. Hosp. Infect.* 74 189–191. 10.1016/j.jhin.2009.10.017 20061059

[B70] Weber D. J. Anderson D. Rutala W. A. (2013). The role of the surface environment in healthcare-associated infections. *Curr. Opin. Infect. Dis.* 26 338–344. 10.1097/QCO.0b013e3283630f04 23743816

[B71] Welsh R. M. Bentz M. L. Shams A. Houston H. Lyons A. Rose L. J. (2017). Survival, persistence, and isolation of the emerging multidrug-resistant pathogenic yeast *Candida auris* on a plastic health care surface. *J. Clin. Microbiol.* 55 2996–3005. 10.1128/JCM.00921-17 28747370 PMC5625385

[B72] Wisplinghoff H. Bischoff T. Tallent S. M. Seifert H. Wenzel R. P. Edmond M. B. (2004). Nosocomial bloodstream infections in US hospitals: analysis of 24,179 cases from a prospective nationwide surveillance study. *Clin. Infect. Dis.* 39 309–317. 10.1086/421946 15306996

[B73] Yildirim M. Sahin I. Oksuz S. Sencan I. Kucukbayrak A. Cakir S. (2014). Hand carriage of Candida occurs at lesser rates in hospital personnel who use antimicrobial hand disinfectant. *Scand. J. Infect. Dis.* 46 633–636. 10.3109/00365548.2014.922694 24953067

